# The AHAWOMEN project: study protocol of a multi-design research for exploring HAPA predictors of exercise in postmenopausal women

**DOI:** 10.1186/s40359-023-01245-9

**Published:** 2023-07-12

**Authors:** Débora Godoy-Izquierdo, Raquel Lara-Moreno, Adelaida Ogallar-Blanco, Juan González, Carlos de Teresa, Nicolás Mendoza

**Affiliations:** 1 Grupo de Investigación Psicología de la Salud y Medicina Conductual (CTS-267), Centro de Investigación Mente, Cerebro y Comportamiento CIMCYC, Universidad de Granada, Campus Universitario de Cartuja s/n, Granada, 18071 Spain; 2 Instituto Universitario de Investigación de Estudios de las Mujeres y de Género, Universidad de Granada, Rector López Argueta s/n, Granada, 18071 Spain; 3 Departamento de Personalidad, Evaluación y Tratamiento Psicológico, Facultad de Psicología, Universidad de Granada, Campus Universitario de Cartuja s/n, Granada, 18071 Spain; 4 Departamento de Psicología Social, Facultad de Psicología, Universidad de Granada, Campus Universitario de Cartuja s/n, Granada, 18071 Spain; 5 Departamento de Obstetricia y Ginecología, Facultad de Medicina, Universidad de Granada, Avda. de la Investigación 11, Granada, 18071 Spain; 6 Centro Andaluz de Medicina del Deporte, Junta de Andalucía, Edificio IMUDS. PT Ciencias de la Salud. Avda. del Conocimiento s/n, 18007 Granada, Spain

**Keywords:** Health action process approach, Postmenopause, Active lifestyle, Health promotion, Adherence, Health-related behaviour change

## Abstract

**Background:**

The postmenopausal period can represent an opportunity for women to improve their health and well-being. The Active and Healthy Ageing in Women during early postmenopause (AHAWOMEN) study aims to identify the key determinants of an active lifestyle among middle-aged women, with a focus on the stages and the social-cognitive variables outlined in the Health Action Process Approach (HAPA) model, a theoretical framework for understanding health behaviour change. We expected that HAPA factors and processes of intention creation (motivational phase) and action adoption (volitional phase) will be significant predictors of exercise initiation and maintenance, supporting both the HAPA tenets and the efficacy of HAPA-based interventions.

**Methods/design:**

This study was approved by the authors’ Institutional Review Committee. Postmenopausal women aged between 45 and 65 years will voluntarily participate. The participants will be allocated to one of three groups: Intervention-Initiators (*n* = 100, random allocation), Control-Sedentary (*n* = 100, random allocation) or Control-Active (*n* = 100, non-random allocation). The intervention group will engage in a supervised exercise programme lasting at least 3 months, supplemented with a HAPA-based intervention for behaviour change. The sedentary control group will not receive any intervention to change their physical activity, while the active control group will consist of women who are already regularly adhering to an active lifestyle. Study variables will be measured at baseline and postintervention phases, as well as at 1, 3, 6 and 12-month follow-ups. The predictors of exercise behaviour in the different phases of the behavioural change process will be explored and compared within and between groups throughout the study. These analyses will help identify the factors that determine the adoption of a healthy active behaviour. Additionally, the effectiveness of the model and the intervention for changing active behaviour will be evaluated.

**Discussion:**

This paper describes the rationale, development and methods used in the AHAWOMEN project. Supporting women who intend to become active can help them to translate their goals into sustainable action. Verifying that the HAPA predictions are applicable to postmenopausal women’s adoption of exercise would provide the basis for designing effective interventions for promoting healthy and active ageing that are also tailored to the experiences of middle-aged women.

**Trial registration:**

ISRCTN16251361. Registration date: 01/06/2023 (retrospectively registered).

## Background

### Ageing, postmenopause, health and lifestyle

In a world faced with the social and public health challenges associated with increased life expectancy, the adoption of a healthy lifestyle has emerged as an essential behavioural resource for promoting and protecting health, enhancing quality of life, preventing and managing illness and addressing the challenges of the ageing process. Lifestyle choices and inequalities in health and disease processes are considered key factors in shaping global health [1]. Thus, it is imperative to prioritise interventions offering appropriate responses to these challenges.

Engaging in regular exercise is essential for middle-aged women to maintain good physical health, improve mental health and overall well-being, increase fitness and quality of life and reduce the risk of chronic diseases related to both ageing and lifestyle such as obesity, cardiovascular diseases, Type 2 diabetes and certain types of cancer [2]. Moreover, exercise has a clear positive impact in terms of psychological and social health outcomes [3]. It is recommended that adults engage in at least 150 min of moderate-intensity aerobic exercise or 75 min of vigorous-intensity aerobic exercise each week, or an equivalent combination of moderate- and vigorous-intensity activity [4]. For maximising health benefits, the international recommendations stress the importance of combining aerobic exercise with muscle-strengthening activities at least two days per week. This will promote cardiovascular and metabolic health, boost immunity and improve muscle strength and bone density as well as other fitness qualities such as balance, flexibility and coordination. Additionally, alternating between aerobic and resistance training can help prevent overuse injuries and provide a balanced exercise routine. These guidelines also emphasise the importance of incorporating physical activity into the daily routine (e.g., using active transportation) and adhering to a planned exercise programme [5]. Organisations such as WHO recommend at least 600 MET minutes per week to attain health benefits, which represents the metabolic rate while physically working reaching the recommended physical activity levels, relative to the resting metabolic rate [5]. These figures fall considerably short of the recommended levels suggested by recent research. Specifically, a minimum of 1200 MET-min/week is now suggested to prevent risk of chronic conditions in adulthood [6] or even higher, that is, ≥ 3000–4000 MET-min/week for individuals to be considered as “moderately active” and ≥ 8000 MET-min/week for “highly active” individuals [7]. Consequently, while daily physical activity is beneficial for health, it is not enough for achieving increased health benefits and should be complemented with moderate-to-vigorous exercise.

Abundant evidence suggests that an insufficient percentage of adults meet the recommendations for a healthy active behaviour, which is more evident as age progresses, especially among women. Despite the benefits of an active lifestyle, women at menopausal age and beyond consistently show the lowest adherence to the recommended levels of physical activity [2]. Specifically, in the southern regions of Spain, some studies estimate that only two-thirds of middle-aged and older postmenopausal women engage in at least 300 min per week of healthy physical activity [8] whereas others report that the total weekly time spent performing moderate-to-vigorous physical activity is 195 min, which is less than 30 min per day [9]. A recent study conducted in Spain, using a representative sample from the general population, suggests that 36% of adults perform < 150 min/week of moderate physical activity, 65% < 75 min/week of vigorous physical activity and 27% did not perform any physical activity at all. Moreover, the study revealed significant differences according to gender (e.g., women perform less vigorous exercise and engage in more moderate activity compared to men) and age, with adult women showing the lowest compliance with international recommendations for vigorous exercise. Moreover, 79% of adult women aged 50–64 years do not comply with recommended levels of vigorous exercise and 75% do not engage in any level of vigorous exercise. However, the statistics show a different picture when light or moderate intensity activity is considered [10]. According to the most recent Spanish National Health Survey in 2018 [11], only 16% of adult women in and after the midlife stage meet the recommended levels of activity necessary for good health.

Therefore, middle-aged women, who experience a multitude of biological, psychological and social changes in their life trajectories when transitioning to or after menopause, while simultaneously navigating the ageing process, represent an important target group for active lifestyle promotion policies. Encouraging an active lifestyle during middle age can have long-lasting effects into older adulthood and provide multi-level benefits to women’s quality of life, beyond the management of menopausal manifestations [12–20]. Such benefits extend to other aspects, including improved functional capacity, cardiovascular and metabolic health, bone mineral density, emotional well-being, sexual functioning, sleep quality and cognitive functioning. An active lifestyle is suggested as a strategy for managing the primary health concerns faced by women during adulthood and older age [21]. However, despite its benefits, exercise is not consistently recommended in clinical practice to middle-aged and older women [22]. Moreover, women encounter difficulties in incorporating regular exercise into their routines due to their multi-tasking, multi-role lifestyles, as well as the socio-spatial processes implicated in physical activities, that are often shaped by gender norms and expectations [23–26]. In addition, those more negatively impacted by midlife-related changes are more likely to engage in unhealthier lifestyles that are maintained over time [27, 28].

### The health-related behaviour change process: from a sedentary lifestyle to regular exercise based on the HAPA model

By identifying behavioural determinants of health and well-being such as an active lifestyle, effective interventions can be developed to increase health, quality of life and longevity [29]. Furthermore, research strongly advises that the designing and planning of interventions aimed at improving physical activity and exercise should be based on behavioural modification theories and behaviour change techniques that have shown to be valid and useful [2]. It has been suggested that interventions for behavioural change should be guided by sound behavioural change theories that help in identifying and modifying the main predictors of behaviour in each of the stages of the change process [30]. This knowledge allows for linking relevant modifiable causal factors of a behaviour to appropriate techniques for behavioural modification [31], and thus for including the specific components that facilitate behavioural change in intervention efforts. A wealth of empirical evidence supports the predictive and transformational power of traditional models of health behaviour change rooted in social cognitive theory (SCT) and its core constructs [32–35]. However, these theories primarily emphasise the motivational determinants of behavioural intentions [36], giving less attention to post-intentional determinants of behaviour adoption and maintenance. Consequently, the percentage of explained behavioural outcomes is low (≤ 30% approx.) [35]. Thus, to provide more successful explanations and effective interventions for behavioural change, theories must incorporate motivational and self-regulatory factors for the short-term initiation and long-term sustainability of behavioural changes.

The Health Action Process Approach (HAPA) model developed by Schwarzer [37–42] integrates and improves on previous social-cognitive theoretical frameworks in describing, predicting and modifying health-related behaviours. It is a hybrid approach that combines the tenets of continuum and stage models of behavioural change process for optimising the explanation, prediction and modification of healthy behaviours. In addition, the HAPA model is a dual-phase model that identifies the determinants of the initiation and maintenance of health behaviour [43]. In the HAPA model, behavioural change is a process involving two main phases (as a stage-centred model) in which several social cognitive predictors exert an influence (as a variable-centred model) on progressing from one stage to the next in the adoption of a health behaviour. It was proposed to particularly address the “intention-behaviour gap” [44], which refers to the fact that forming good intentions to act does not necessarily translate into action [45]. This observation helps explain why a significant proportion of individuals who have the goal of adhering to a healthy behaviour fail to translate their intentions into adopting the desired behaviour. Thus, one of the main contributions of the HAPA model is to distinguish between two stages or phases, each comprising a set of constructs and processes that determine behavioural enactment: an initial motivational phase that leads to the creation of a behavioural intention, and a subsequent volitional phase that leads to the implementation of an actual health behaviour. Phase-specific self-efficacy beliefs, planning and action control, depending on the stage in which the individual is involved in, namely non-intention, intention, action and disengagement, determine the progression in the change process. Stages and variables should be considered to optimise the advancement in behaviour change until a regular and sustainable habit is created.

Pre-intentional, motivational factors play a significant role in shaping intention. These factors include perceived vulnerability, which refers to the awareness of a potential threat to health resulting from a current unhealthy behaviour; outcome expectations, which involve anticipating the beneficial consequences of an alternative healthier behaviour; and, most notably, perceived action self-efficacy, which is the belief in one’s own ability to initiate the healthy behaviour. Higher levels of action self-efficacy increase the likelihood of initiating the new behaviour. These motivational factors are the prerequisites for setting behavioural goals (i.e., intentions) and provide the motivational resources that ultimately translate intention into a new behaviour [39].

A major contribution of this model is its emphasis on the post-intentional phase of behavioural change, which incorporates the self-regulation elements necessary for successfully translating a proposed goal into an actual action (initiation subphase) that is sustained over time (maintenance subphase). Engaging in a sustainable healthy behaviour in daily life requires constant self-regulatory efforts that involve establishing and maintaining behavioural goals, execution planning and managing one’s behaviour [37]. Consequently, specific self-efficacy beliefs and planning as well as action control were introduced as volitional mediators in the intention-action path and the long-term adherence to action. Specifically, action planning (if–then plans, also known as implementation intentions) and coping planning (if barrier-then coping plans) constitute a prospective, preparatory self-regulatory skill involving the strategic elaboration of organised sequences of actions, detailing the performance of behaviour in anticipated facilitatory situations, or an alternative behaviour in the face of obstacles or barriers. Action planning involves creating a detailed plan that includes specific instructions on how, when, where and other relevant parameters for performing an action, while coping planning entails developing alternative plans that anticipate barriers and obstacles that may be encountered and specify strategies for overcoming them. Planning is proposed to work in conjunction with maintenance self-efficacy, which is the belief in one’s ability to sustain the intended behaviour by effectively dealing with obstacles during the behavioural maintenance phase, and recovery self-efficacy, which refers to the perceived confidence in one’s ability to recover from setbacks and maintain adherence to the new behaviour. These factors contribute to facilitating automaticity and efficacy in behavioural enactment [37, 39].

Thus, the HAPA approach provides a parsimonious yet synergistic framework that captures the main proximal factors and processes involved in the adoption and maintenance of health-related actions.

The HAPA model has been successfully applied to the adoption and maintenance of a wide range of health-related behaviours (see the meta-analysis with meta-analytic structural equation modelling by Zhang et al. [43]) including physical activity and exercise. Gholami et al. [46, 47] synthesised and meta-analysed cross-sectional, longitudinal and experimental/intervention research investigating the main associations between HAPA constructs and physical activity and exercise behaviour. The summary effect sizes generally supported the model’s predicted associations across samples. The meta-analytic study by Zhang et al. [43] has provided further support for the HAPA direct and indirect predictions in the context of physical activity and exercise. Choi et al. [48] meta-analysed cross-sectional and longitudinal studies on active behaviour (excluding experimental/intervention studies), finding a medium overall effect size (0.28) for the impact of HAPA variables on physical activity. This effect size is comparable to that of the combined influence of the main social-cognitive theory-based constructs including self-efficacy, outcome expectations, goals/intention and self-regulation [35]. It is also consistent with the average efficacy of theory-based interventions for promoting an active lifestyle [30] and the most commonly used behaviour change techniques for adopting active behaviours [49] and maintaining them over a 6 to 15 month period [50] in adult and elderly populations. The findings of all these meta-analyses generally support those of similar previous meta-analyses on the associations between social cognitive determinants including intentions, stage-specific self-efficacy beliefs, action and coping planning and active behaviour [35, 51–53]. This information serves as a basis for designing high-quality theory-guided interventions for the promotion of an active lifestyle.

The HAPA model has been tested with a wide range of populations, sociocultural backgrounds and health behaviours, and has demonstrated its universality and stability across different scenarios [37, 43, 54]. Nevertheless, the existing evidence also points to certain differences based on gender, age, type of behaviour or health status. While the model is applicable to all individuals, some findings indicate that certain adjustments could enhance its applicability and power. For instance, some evidence indicates that the HAPA model is particularly applicable to middle-aged and older adults in predicting an active lifestyle [55] and interventions tailored to specific age groups [56] are expected to be more successful than one-size-fits-all approaches. Other research indicates that adult women utilise different resources throughout the change process compared to men (e.g., higher planning, lower social support) [57–59], which could indicate that women face specific circumstances that make it more challenging to adopt a new habit and receive acceptance from their social contexts. As a result, they may be more likely to engage in less physical activity and require tailored interventions that are adapted to their particular realities, experiences, resources and opportunities. While initial meta-analytic research found minimal effects of age, gender or sample type as moderators of the associations between HAPA constructs and exercise behaviour [46], the findings also revealed high heterogeneity. More recently Choi et al. [48] reported that effect sizes for the predictions based on the HAPA tenets were larger in the healthy participants compared to patients, men compared to women and younger individuals compared to older ones. These findings are congruent with the notion that healthy people, men and younger individuals are more likely to engage in physical activity. Zhang et al. [43] concluded that the population characteristics that were found to have minimal differences did not deviate significantly from the tenets of the HAPA model. However, these findings stress the relevance of understanding the resources, opportunities and barriers involved in self-management and self-regulatory processes that some populations — such as middle-aged women who have gone through the menopause — may encounter when attempting to make behavioural changes. The authors thus recommended that interventions be tailored to the specific circumstances of the targeted individuals. All these findings stress the relevance of investigating the gender-, age- and health status-based specificity of the process of behavioural change for health-related actions and the associated factors and dynamics involved, which can inform the development of appropriate interventions [60].

Research on the applications of the HAPA model to exercise behaviour is scarce among women. The model has primarily been tested in reproductive status-related conditions such as pregnancy [61], while research focused on middle-aged women has included breast cancer survivors [62, 63]. This research has therefore been conducted in women with reproductive (e.g., premenopausal women) or health conditions (e.g., severe or chronic illness) that are physiologically, psychologically and socially unaligned with a healthy postmenopausal status. Barg et al. [64] conducted a study involving inactive and generally healthy 40–65 yr old women. The participants received an intervention with information on physical activity levels for cancer prevention, the effect of which was considered to be “negligible” (p. 4) by the authors. This study aimed to examine changes in inactive behaviour and the motivational and volitional predictors based on the HAPA model over a 12-week period. Malaijerdi et al. [65] carried out a study targeting inactive women aged 30 to 60 years, implementing a brief educational intervention consisting of three one-hour sessions focused on exercise and health to improve HAPA motivational predictors of intention (motivational phase). They assessed HAPA predictors and intention before, immediately after the intervention and one month later and compared these with a control group of similar women not receiving such an intervention. Joveini et al. [66] reported the findings of those participants who subsequently developed the intention to exercise and who received a HAPA-based intervention aimed at offering them resources for planning the new behaviour and coping efforts as well as maintenance and recovery self-efficacy (volitional phase skills). The participants were followed for three months (HAPA constructs) and six months (behaviour). Evers et al. [67, 68] conducted a study with inactive older women in their 70 s to 90 s to investigate the short-term (six weeks), medium-term (10 weeks) and long-term (20 weeks) adherence to a supervised 6-month exercise programme. The authors offered an (assisted vs. non-assisted) intervention focused on coping planning (six weeks after starting the programme) and explored HAPA volitional predictors of adherence. Although all these studies started with and followed sedentary women, some lacked controls for comparing the influences of HAPA constructs [64] and others compared subgroups of women receiving or not receiving a HAPA-based intervention [65] or the effects of HAPA-based interventions with different delivery formats [68]. Consequently, to develop effective intervention strategies for improving physical activity among women after menopause, it is necessary understand the determinants of an active lifestyle in this population.

### The AHAWOMEN project: aims and hypotheses

The Active and Healthy Ageing in Women during early postmenopause (AHAWOMEN) project focuses on the key issue of the applicability of the HAPA model to exercise behaviour in postmenopausal women. A deeper understanding of exercise determinants in this population will allow designing appropriate interventions to promote exercise, helping women to successfully navigate the process of behaviour change. The project explores the utility of the HAPA model in combination with other psychosocial and biomedical correlates of exercise in this population, while being sensitive to the unique experiences, needs and opportunities of women in their middle age. Specifically, the objective of the AHAWOMEN project is to explore the main predictors of healthy exercise in postmenopausal women including fitness, cardiometabolic health, mental well-being, health-related quality of life, menopausal factors (e.g., frequency, intensity and burden of manifestations and symptoms) and particularly the social-cognitive constructs outlined in the HAPA model at every stage of the process of behaviour change while controlling for personal conditions (e.g., age, education level, occupational status, income, marital status). The ultimate aim is to support both theory- and evidence-guided interventions for the promotion of an active lifestyle among postmenopausal women.

Consequently, the central objective of the AHAWOMEN project is to examine the applicability of the HAPA model in explaining and predicting health-related behaviours such as exercise within this population, while taking into account the women’s own personal and social conditions, experiences, resources and opportunities.

To test the HAPA tenets in middle-aged postmenopausal women while filling the abovementioned gaps in the literature, the main aim of this study is two-fold. First, we aim to explore the influence of the social-cognitive variables relevant at each stage of the process of behavioural change based on HAPA predictions, and second, to test the goodness of fit of the model for exercise behaviour among women in early postmenopause (up to ten years after menopause). Specifically, the sub-aims of the present study protocol are the following:

1. To explore the HAPA constructs at every stage of the change process in new exercisers, that is participants adhering to exercise practice while simultaneously receiving an intervention based on the HAPA model (Intervention group). These will be compared with regularly active (active control) and non-active (sedentary control) postmenopausal women, all with different socio-demographic, personal and clinical characteristics. Consequently, this sub-aim has the following specific objectives:
  - to determine changes in the HAPA variables in the intervention group throughout the study phases, and in this regard:
    H1: we hypothesise that there will be a significant improvement in both intentional-motivational and postintentional-volitional variables in the participants in the exercise programme supplemented with the HAPA intervention.
  - to compare the intervention, sedentary and active groups across the motivational and volitional phases of the HAPA model, and in this regard:
    H2: we hypothesise that, at baseline, non-active women (i.e., sedentary and intervention groups) will show similar HAPA determinants of exercise intention, while the active controls will obtain more positive results in the HAPA motivational constructs, and
    H3: we hypothesise that, at post-intervention and subsequent follow-ups, the new exercisers and the active women will show similar HAPA self-regulatory skills of the volitional-actional stage but will differ significantly from the sedentary women.
2. To explore whether the HAPA constructs at the different stages of the process of behavioural change — i.e., non-intention, intention, action and disengagement — and phases of the process, that is, motivational (preintention) and volitional (intention-action-adherence), predict the adoption of exercise behaviour among postmenopausal women. In this regard:
  H4: we hypothesise that while the HAPA model will be applicable to this population, supporting its main predictions, adjustments in the model predictions will be necessary to optimise its explanatory power, considering the experiences, resources and opportunities of middle-aged women, and
  H5: personal (i.e., fitness status, physical and mental health status and sociodemographic variables) and clinical factors (i.e., menopausal manifestations and menopause-related quality of life) will intersect with HAPA variables in the prediction of exercise behaviour and behaviour change, supporting the existence of phenomena that are pertinent and exclusive of middle-aged women and are grounded in their experiences, realities and needs.

## Methods

### Study design

**Fig. 1:**
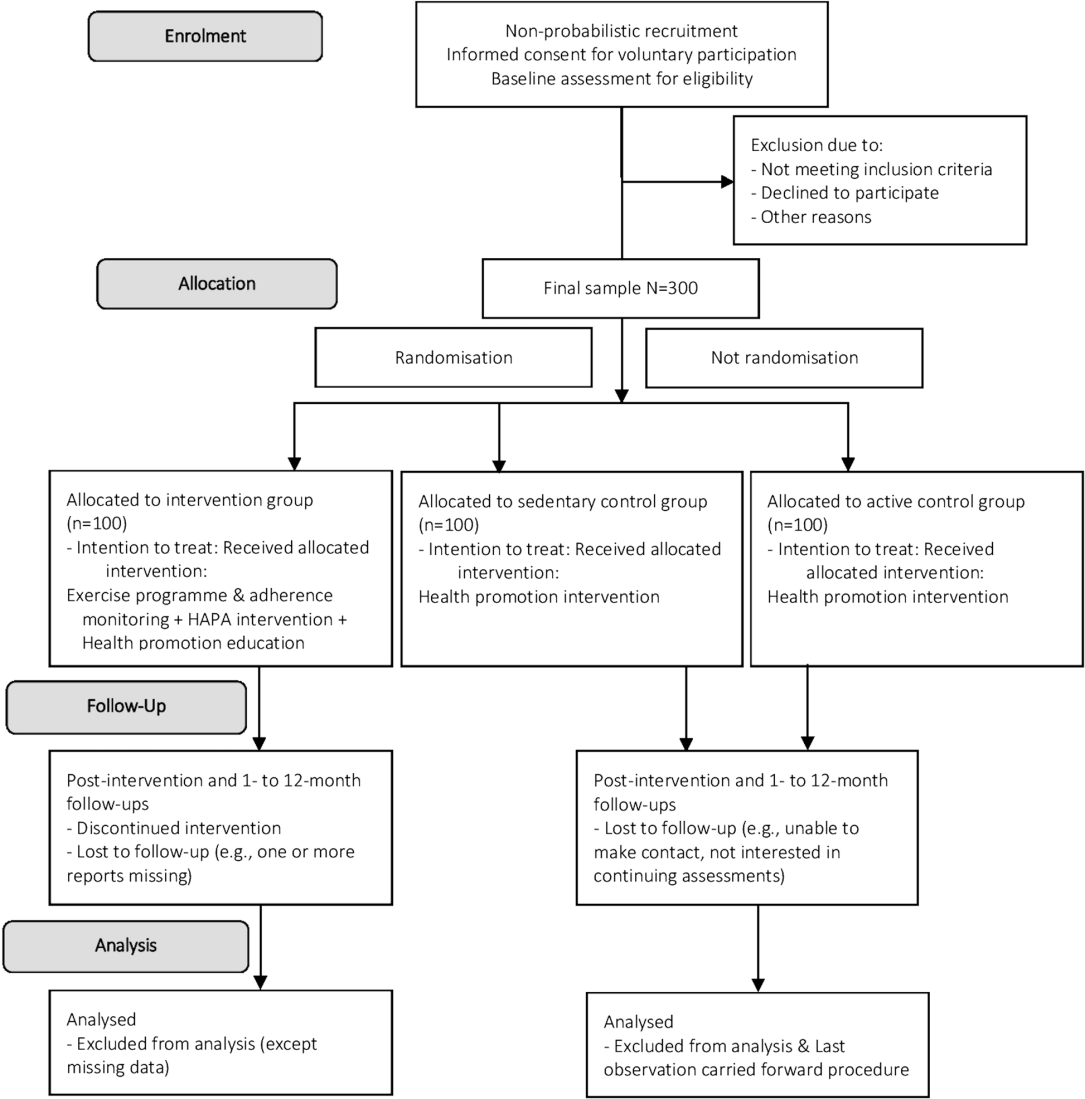
Flowchart with the stages of the research activity and the recruitment and study group assignment

This project is designed as a longitudinal prospective, multi-design study, incorporating both a randomised controlled study (for intervention and sedentary participants) and a non-randomised controlled study (for intervention/sedentary and regular active participants). The study will utilise between-group (intervention, sedentary and active participants) and within-subject measures (baseline, post-intervention and 1- to 12-month follow-ups) (see Fig. 1). The Intention-to-treat methodology, considered the gold standard for RCTs [69, 70], will be implemented to ensure that data from the participants in each assessment phase fit with the inclusion criteria of each study group, with the aim of preventing interferences caused by unexpected changes over time in their assigned condition (e.g., an active women withdraws regular practice due to injury; or a sedentary woman is motivated to autonomously become an exerciser). Moreover, this approach will help to mitigate the potential reduction sample size and loss of statistical power resulting from participant attrition.

The SPIRIT recommendations for the design and implementation of interventional trials [71] were followed in the design of the project. The CONSORT guidelines for reporting RCTs will be observed for communicating the findings [72].

### Participants and eligibility criteria

The participants will be 300 postmenopausal women aged 45 to 65 years living in Granada, a city in the South of Spain with a population of > 230,000, as of 2023 [73]. Participants will be recruited using non-probabilistic, convenience procedures (see Procedure section).

Inclusion criteria will be: 1) being postmenopausal women between 45 and 65 years old; 2) with no menses for 12 months or more and up to 10 years after menopause, i.e., early postmenopause based on Stages of Reproductive Ageing Workshop STRAW + 10 criteria [74]; 3) able to speak and read Spanish with proficiency; and 4) able to provide informed consent for voluntary participation. Additionally, inclusion criteria for the intervention/sedentary control groups are: a) being sedentary (having not engaged in moderate-to-vigorous exercise on a regular basis, independent of daily physical activity, e.g., walking for transportation) for at least the 12 months prior to the beginning of the study, as screened with the self-report described below; b) able to exercise, i.e., in good health that allows them to exercise, based on medical criteria (see below); and c) willingness to be randomised to intervention/non-intervention (waiting list) conditions. For the active control group, these criteria are: i) exercising on a regular basis reaching international recommended levels for health promotion for at least the last 12 months, as screened with the self-report described below. In addition, exclusion criteria are: 1) use of hormone therapy in the three months before recruitment; and 2) suffering from any physical or mental disease that would seriously deteriorate functioning, impede participation in exercise based on medical criteria or introduce bias in responses. For the active participants, an athlete (i.e., competitive sport) of any performance level will be excluded. Selection criteria will be examined after recruitment to check eligibility and proceed with the study group assignment.

After enrolment, participants will be assigned to one of three study groups by randomisation and non-randomisation, depending on the study condition. The groups are as follows: 1) Intervention-initiators (participants who will engage in a supervised exercise programme complemented with a HAPA-based intervention, *n* = 100); 2) control-sedentary (non-active women who will maintain their sedentary lifestyle and will not receive any intervention for promoting behavioural change, *n* = 100) and 3) control-active (women with a regular and sustained exercise habit, *n* = 100).

The sample size has been calculated to include 80 participants per group, considering an *alpha* error of 0.05 and a *beta* error of 0.2, for independent groups and continuous variables, i.e. mean comparisons, with estimated mean values based on previously published studies [64, 65, 68], to ensure sufficient statistical power to detect a moderate treatment effect in a clinical study (Clincalc®). To account for potential dropouts, we anticipate a 20% dropout rate, increasing the sample size up to 100 participants per group. 

Figure 1 shows a flow chart of the study participants, including the selection process.

### Measures

Measurements will be taken at baseline, post-intervention and at 1-, 3-, 6- and 12-month follow-ups. This comprehensive assessment will enable the exploration of the factors that predict the initiation and maintenance of exercise behaviour, as well as the stages of the behaviour change process. 

#### Personal data (baseline)

Participants will complete a socio-demographic, clinical and lifestyle data form including information on age, educational level, employment status, occupation, average household income, marital status, number of children, family caring roles and current major stressful life events.

Moreover, the participants’ personal medical history will be obtained through clinical interviews conducted by the gynaecologists collaborating with the research team [75]. Information will be gathered on: a) Gynaecological antecedents including age at menarche, main features of menstrual cycles (e.g., regularity, dysmenorrhea), age at final menstrual period, time since menopause, obstetric history on gravidity and parity (GTPAL, G = gravidity, T = term deliveries, P = preterm deliveries, A = abortions or miscarriages, L = live births), age at first and last pregnancy and natural lactation; b) life-course, recent (last 12 months) and current gynaecological and general main diseases and surgeries, recent (last 12 months) and current drug treatments (particularly corticosteroids), previous and current use of hormone therapy, previous bone fractures and previous bone densitometries; c) regular gynaecological (breast and pelvic) screenings and hormone blood tests; and d) family history (especially bone fractures, cardiovascular diseases and gynaecological cancers).

Lifestyle will be explored through an *ad-hoc* self-report survey on health-related behaviours in key areas of women’s health. These areas include physical activity and exercise, nutrition, sleep, leisure, substance use (e.g., alcohol use and smoking), sexual relationships, emotional self-regulation, social relationships and support, self-protection (e.g., sun protection), health responsibility (e.g., regular medical screenings) and life appreciation and self-actualisation (e.g., life satisfaction, meaning and purpose in life) based on previous questionnaires [76].

#### Active behaviour (all assessments)

Active lifestyle will be assessed with the short form of the International Physical Activity Questionnaire (IPAQ) [77], Spanish version [78]. The IPAQ is a widely used self-report instrument for assessing physical activities that people carry out as part of their daily routines. These include activities related to transportation, occupation, household chores and recreation. This questionnaire enables the estimation of total light, moderate and vigorous physical activity and exercise, as well as the amount of time spent sitting during the last seven days. The data gathered using the IPAQ can be used to determine indicators of volume of physical activity and energy expenditure (e.g., METs-min/week). These indicators can then be compared against recommended levels of healthy activity. The psychometric properties of the IPAQ have been demonstrated in the Spanish population [78, 79]. The information collected with the IPAQ at baseline will be used to assign the participants to the study groups, as well as to assess activity levels throughout the study.

#### HAPA constructs (all assessments)

To assess the HAPA constructs, the following measures will be used, based on published guidelines and existing measures [37, 80, 81], as well as studies conducted on physical activity and exercise, particularly among women [64, 65, 68], adapting their content to postmenopausal women.

At baseline, the HAPA constructs of the motivational phase will be measured as indicators of the motivation for exercise and predictors of the creation of an intention to engage in a healthy and active lifestyle. The following constructs will be evaluated:

- Absolute (personal risk) and relative (compared to an “average” woman of the same age and health condition who has transitioned the menopause) risk perceptions. Each perception will be assessed using five items that include the main physical and psychological manifestations of menopause and ageing processes (e.g., hot flushes and night sweats, sleep disturbances, low mood, mental slowness). Some items will also address conditions related to an active lifestyle (e.g., fatigue, weight gain, body image, severe health conditions). The statements are based on current evidence on health risks linked to menopause and the ageing process [82]. Participants will respond on a scale ranging from 1 (very unlikely) to 7 (very likely), and average scores will be calculated to obtain subtotal and total scores.
- Outcome expectations of adhering to regular exercise. These will be assessed with 15 items, 12 of which assess positive outcomes — i.e., expected benefits — including three main types of outcomes: 1) personal enjoyment and well-being (e.g., “I will feel better afterwards”, “I will feel satisfied with myself and proud of myself”), 2) management of menopause- and ageing-related changes and health (e.g., “I will manage better the changes and manifestations of my menopause”, “My quality of life will improve”) and 3) social recognition and support (e.g., “Other people will appreciate my willpower”, “I will be appreciated by others for that”), thus incorporating both intrinsic and extrinsic motivational regulations [83] that can drive exercise behaviour among middle-aged and older women [84, 85]. Negative outcomes — i.e., problems and difficulties derived from exercise — will be assessed with three items including feelings of discomfort resulting from doing something the participant does not like, time organisation difficulties and family financial challenges due to spending money on training and equipment (e.g., “It will be a burden on my financial situation”). Responses will range from 1 (not at all true) to 4 (completely true). Average scores will be calculated to obtain subtotal scores, and the difference between these scores will serve as an indicator of the balance between positive and negative outcomes, with positive scores suggesting a higher presence of benefits compared to costs.
- Action (task) self-efficacy. This assessment will include two components: a) Four items will assess motivational self-efficacy, which refers to an individual’s belief in their ability to adopt an active lifestyle, be physically active in daily life and engage in regular exercise at the minimum and optimal recommended levels (e.g., “I’m confident in myself for… doing vigorous-intensity exercise at least 3 times a week for 30 min or more”). b) Four items will assess preactional or task self-efficacy, which relates to an individual’s belief in their ability to overcome barriers and difficulties when immediately starting regular exercise (e.g., “I’m sure I can start exercising immediately… even if I have to reconsider my views on exercise”). Participants will respond on a scale ranging from 1 (not at all true) to 4 (completely true). Average scores are calculated to obtain subtotals for each component and a total score.
- Intention. Behavioural intentions for the following weeks will be assessed using a multi-habit measure consisting of 14 items. These items will correspond to various aspects of adopting a healthy lifestyle, such as improving/adopting healthy behaviours (e.g., healthy eating and weight control, emotional regulation, family and social relationships, sleep, leisure) and reducing/quitting unhealthy habits (e.g., tobacco or alcohol use), menopause-related health management (e.g., taking proactive steps for personal health, commitment to medical examinations), as well as increasing daily physical activity (1 item) and engaging in regular exercise at minimum (1 item) and optimal levels (1 item) (e.g., “I intend to… do at least 150 min per week of at least moderate intensity exercise”). Participants can respond on a scale ranging from 1 (don’t intend to at all) to 7 (strongly intend to). Average scores will be calculated to obtain a total score for 1) intention to adopt a healthy lifestyle, 2) intention to adopt an active lifestyle and 3) intention to engage in regular exercise at the optimal recommended levels.

In the post-intervention and follow-up phases, the HAPA constructs of the volitional phase will be measured as predictors of the translation of the intention into action, both at the initial adoption of the active behaviour and the medium- and long-term adherence to such behaviour. These constructs will be:

- Outcome perceptions. These refer to an individual’s perceptions of the actual results achieved by engaging in exercise and will be assessed with the same measure used for assessing outcomes expectations at baseline. However, the wording of the items will be modified to assess perceived outcomes (e.g., “I have felt better afterwards”). The same indicators as those used for the baseline measure will be obtained for this assessment.
- Action planning for autonomous regular exercise. Action planning refers to the development of specific, organised behavioural plans for exercising after the intervention. It will be assessed using five items asking about the details of the exercise plans, including what, how, when, where, how often and with whom the exercise will take place once the supervised programme has finished (e.g., “I already have a structured plan detailing… the duration, intensity and frequency of my exercise routine”). Participants will respond on a scale ranging from 1 (not at all true) to 4 (completely true). An average score will be calculated based on these responses.
- Coping planning for maintaining regular exercise. Coping planning involves developing specific and organised behavioural plans to manage obstacles and setbacks. It will be assessed using five items asking about the specific strategies that participants have in place to overcome challenges such as interference with daily/weekly training plans, missing training sessions temporarily, staying committed to their exercise intentions when facing difficulties, remaining dedicated to exercise and being vigilant about situations that could lead to lapses and relapses (e.g., “I already have a structured plan detailing… what to do if I miss one training session”). Participants will respond on a scale ranging from 1 (not at all true) to 4 (completely true). An average score will be calculated based on these responses.
- Maintenance or coping self-efficacy for regular exercise. It will be assessed with 11 items that address the main obstacles middle-aged women can typically encounter when engaging in regular exercise. These obstacles include difficulties in creating the exercise habit (requiring multiple attempts or taking a long time to generate the habit), not experiencing immediate improvements in health and well-being, low mood, tiredness, lack of social support or having a partner or family members with a sedentary lifestyle (e.g., “I have confidence in myself to…stick to my exercise routine even if I don’t experience an immediate improvement in my menopause symptoms”). Participants will respond on a scale ranging between 1 (not at all true) and 4 (completely true). An average score will be calculated based on these responses.
- Recovery self-efficacy for managing lapses and relapses. It will be assessed with 14 items that address the main barriers middle-aged women are likely to encounter when re-engaging in exercise. These barriers include anticipating and managing difficulties in maintaining the habit (e.g., postponed plans), not feeling mentally prepared or motivated, not experiencing improvements in well-being or menopausal symptoms, being in low mood, feeling tired, lacking social support or having a partner with a sedentary lifestyle (e.g., “I am sure I can resume my exercise routine even if… I have already taken a break for several weeks”). The participants’ responses will range between 1 (not at all true) and 4 (completely true). An average score will be calculated based on these responses.
- Behavioural intentions for the subsequent weeks/months will be assessed at post-intervention and follow-up phases with the same measure as that used in the baseline phase. The same indicators will be obtained.

#### Health and Well-being (all assessments)

Several objective and subjective measures regarding health and well-being will be also administered to complete assessments of correlates of exercise. These measures will include generic [86, 87] and menopause-related health-related quality of life [88], perceived health status (single-item indicator, 0 = the worst possible to 10 = the best possible) [89], lifestyle health-related behaviours [76], motives, expected benefits, beliefs and attitudes towards exercise (*ad-hoc* self-report adapted to middle-aged women based on previous published questionnaires [90], subjective well-being [91] and cardiometabolic health indicators. The latter consist of both structural (e.g., objective BMI and body composition using bioelectrical impedance analysis) and functional indicators (e.g., heart functioning, aerobic aptitude index) as well as fitness indicators [92]. These measures are fully described elsewhere [93, 94].

**Fig. 2:**
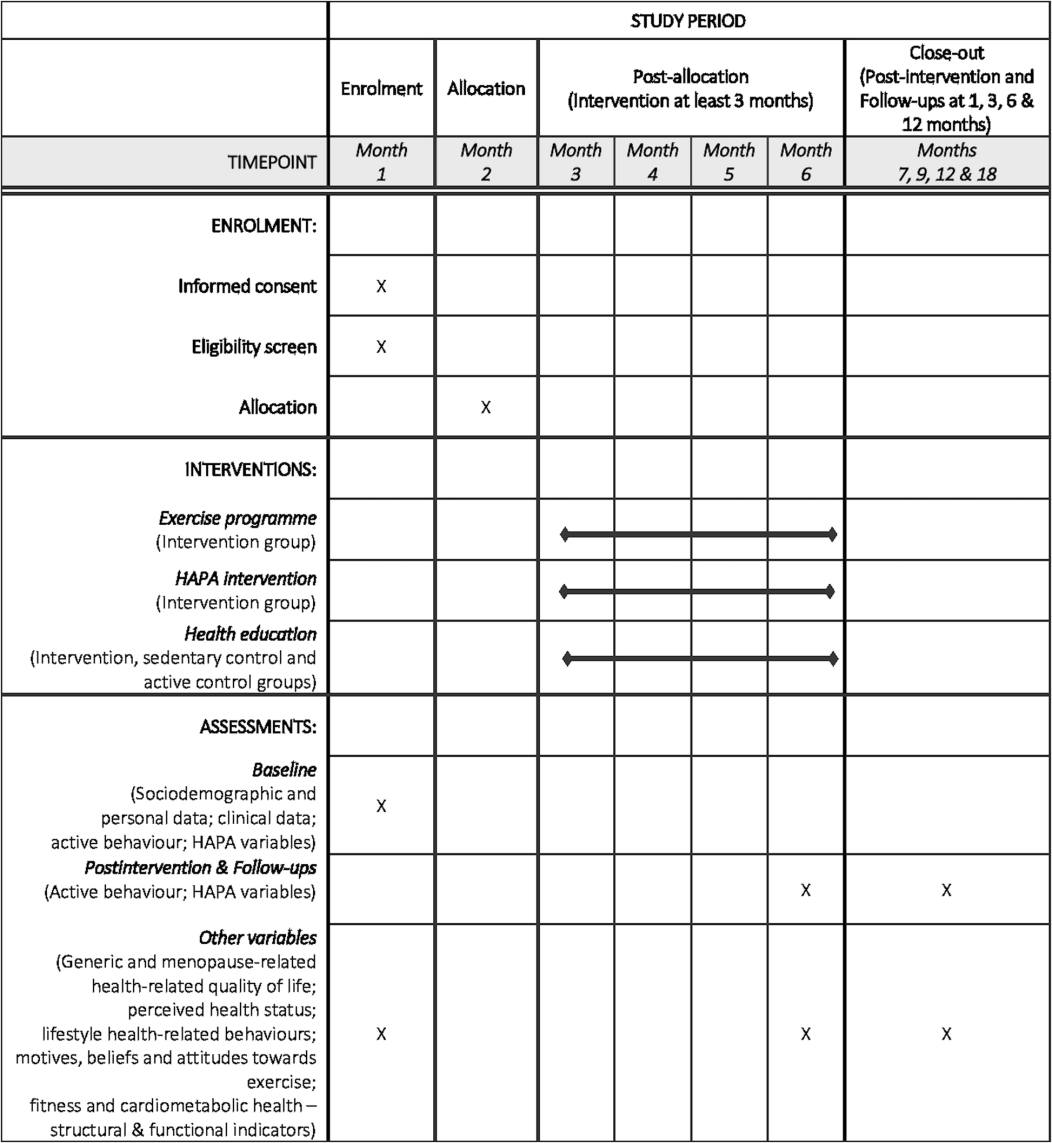
SPIRIT timeline of enrolment, interventions and assessments

By measuring these predictors, correlates and outcomes, we will gather the required information on personal and interindividual differences across multiple domains of variables related to exercise. Additionally, we will gain insights into contextual factors that are likely to shape health-related and active behaviours among postmenopausal women. Figure 2 provides an overview of all measures and the corresponding time points for data collection throughout the study.

### Procedures

The study will be publicised through several channels (e.g., media advertisements, online social networks, institutional e-mailing, gynaecology outpatient clinics, women’s associations, gyms, fitness clubs, community and university exercise programmes, neighbourhood associations, other community resources, snowball sampling) to recruit a wide sample of postmenopausal women interested in participating, along with information on how to contact the researchers (an e-mail and a telephone number). Once potential participants contact the research staff, they will be required to attend an informative session where they will receive detailed information about the general goal and procedures of the study, their rights and tasks as participants and topics such as the confidentiality of their responses and the exclusive use of data for scientific purposes. During this meeting, participants will be encouraged to express any doubts or concerns and to consult with the research staff. After agreeing to continue with enrolment, participants will be asked to sign an informed consent form. A research assistant will then contact each participant to schedule two lab visits for the first assessment phase, which will include a face-to-face clinical interview, the self-report-based evaluation conducted through an online survey implemented using an open access resource such as Limesurvey (Limesurvey GmbH, Hamburg, Germany) [95] and the remaining measures that need to be completed (e.g., weight, fitness tests). Baseline measurement will be conducted with all the women to screen eligibility criteria and collect pre-intervention data.

Upon meeting the eligibility criteria, the final study sample will be allocated to an intervention group receiving the intervention (*n* = 100), a control group of sedentary women (*n* = 100) and a control group of regularly active women (*n* = 100). The non-active participants will be randomly allocated to two groups: the intervention group and the sedentary control group, making this a randomised controlled arm of the study. This random allocation will be performed by, for example, computer-generated random numbers. The sedentary control group will be considered a waiting list group. On the other hand, women who already engage in regular exercise will be assigned to the active control group. Since these women are already active, this will be a non-randomised controlled arm of the study.

### Intervention

A three-component intervention will be offered, which includes:

1. A multicomponent, supervised exercise programme with a 3-month (12 weeks) minimum duration will be offered to the intervention group. The exercise programme will consist of three 1-h weekly sessions on alternate days. Participants will be able to choose from several schedules available in both the morning and afternoon periods. The programme will be designed and implemented by experts in Exercise and Sport Sciences and Sport and Exercise Medicine, following international recommendations for exercise in middle-aged women [96, 97]. The programme will include training for aerobic cardiorespiratory fitness, muscle resistance, as well as other fitness functions including flexibility, coordination and balance. Hence, it will meet the required conditions in terms of activity type, duration, frequency and intensity parameters and principles and methodology of training for a healthy, safe, successful and enjoyable practice for adult women, thus being suited to their characteristics and needs for maximising benefits while minimising risks. The programme will be implemented in supervised group classes conducted in a gym facility (e.g., in the university campus). To assess adherence to the programme, objective tracking measures will be utilised, including attendance monitoring. All these features are designed to optimise participation as well as outcomes. Additionally, a health insurance policy will be put in place to cover any potential risks and provide necessary medical assistance if required.
2. A simultaneous intervention focusing on self-management of behavioural change, based on the tenets of the HAPA model, will be offered throughout the duration of the exercise programme. This intervention has been designed based on previous HAPA-based interventions [98–103]. To summarise, the intervention will provide information on the benefits of an active lifestyle and exercise, specifically for postmenopausal women, addressing risk perceptions and outcome expectations while facilitating the creation of an intention (Session 1). The participants will be also educated on the key factors for adopting and maintaining an active behaviour, such as identifying barriers and facilitators, basic self-monitoring and self-management skills for action regulation (e.g., self-monitoring, self-reinforcement, setting a daily time schedule, emotional regulation, seeking social support and reinforcement from significant others); these elements contribute to improving motivational action self-efficacy (Session 2). The subsequent 3 to 5 sessions will focus on motivational determinants of exercise initiation, including goal setting (behavioural intention), action planning and pre-actional/task self-efficacy to improve motivation and action initiation. Sessions 6 to 9 will address volitional determinants of maintaining adherence, covering self-regulatory skills including action and maintenance plans for autonomous exercise, maintenance and coping self-efficacy and relapse management (i.e., risk identification, functional responses and coping skills training). A final session is devoted to summarising the personal progress of the participants with this intervention. The aim of this intervention is to ensure that women not only adhere to the supervised exercise programme offered within the study, but mainly to promote long-term, self-managed adherence to the newly established lifestyle beyond the completion of the supervised exercise programme. To achieve this, the adherence promotion intervention will be delivered during the entire implementation of the exercise programme, divided into two phases according to the process of behavioural change. During the first phase of the programme, the focus will be on addressing issues pertaining to initiation and short-term maintenance. Researchers and implementers will play a central role in providing external regulation and support as a strategy to facilitate adherence. During the second phase of the programme, the emphasis will shift toward long-term maintenance. Building on the knowledge and skills acquired by the participants, the intervention will promote the transition to self-regulation and the development of intrinsic motivation based on enjoyment and personal growth.

During the interventions described in 1) and 2), attendance to the exercise programme will be monitored by the trainer. Regular updates will be provided to the participants by the researchers and will serve as a basis for working with the behavioural change intervention.

3) A health education and promotion intervention (15 60-to-90 min. sessions) designed and implemented by experts in women’s health, will also be offered. This will focus on providing information and recommendations regarding a healthy lifestyle during postmenopause. The issues covered will include healthy eating and weight management, sexuality and intimate relationships, family and social support, social activities, emotional self-regulation, body appearance management and body image, unhealthy habits such as tobacco and alcohol use and other health-related behaviours (e.g., sleep, leisure time habits). This intervention will also be offered on a voluntary basis to the participants in the control groups to ensure equal treatment among the study groups in terms of lifestyle after menopause (except for active behaviour). Health education is typically provided as standard care in menopause and postmenopause (e.g., EMAS Consensus Statement [82].

After the multi-component intervention, measurements will be repeated at post-intervention and follow ups. All the measurements will be conducted to ensure that the evaluators are blinded to the participants’ allocation to the study groups (e.g., online survey). 

To encourage adherence to the procedures, various strategies will be implemented, including the provision of regular feedback on attendance to the exercise programme and sending reminders to participants regarding the assessment phases. Additionally, in cases of non-compliance, motivational inquiry will be conducted through open and supportive dialogue to address the underlying reasons and to ensure compliance. Nevertheless, disengagement can offer interesting information, and the non-adherent participants will not be excluded from the study or analyses. 

Figure 1 illustrates the stages, planning and distribution of the research activity as well as the flow of the current study procedures. The timeline of the procedure is described as a SPIRIT figure in Fig. 2.

Ethical approval has been obtained from the Ethics Committee on Human Research of the University of Granada (Spain), reg. B-CTS-342-UGR20 (2021–23), on November 17, 2021. Written informed consent will be obtained from all the participants. All procedures will be conducted in accordance with the ethical principles for research involving human subjects detailed in the Declaration of Helsinki and updates. Study participation will be voluntary. Reasons for withdrawal may include exercise-related injuries, significant changes in health status, hormone therapy recommendation or the participant’s personal request to withdraw or refuse consent at any time during the study.

The study protocol has been pre-registered at BMC ISRCTN registry (ISRCTN16251361).

### Data management and analyses

Data containing personal information will be securely stored in either a locked cabinet or on password-protected devices in the researchers’ laboratory. Each participant will be identified using a unique number code, and the anonymised data in databases will be used for analytical purposes. The databases will be stored securely.

Statistical analyses will be conducted using appropriate statistical packages (e.g., SPSS 28.0 and AMOS 22, IBM SPSS Inc., Chicago, IL). Once the database has been downloaded and thoroughly cleansed, preliminary and exploratory data analyses will be conducted. The aim of these analyses will be to detect potential errors in the data, missing data and data distribution. This will also help to verify data assumptions and select appropriate statistical tests. Missing data will be treated with pairwise deletion. Alternatively, when appropriate and feasible (e.g., for follow-up data from control participants since no change in outcomes is expected if their condition remains unchanged), the last observation carried forward procedure will be used for the imputation of missing data [104, 105]. Univariate (boxplot) and multivariate outliers (Mahalanobis distance D^2^ and cut-off values for a conservative significance level of *p* < 0.001) [106] will be identified before analyses. To ensure higher internal, ecological and external validity, the main confounding variables will be controlled for in the analyses (e.g., sociodemographic variables by blocking or matching, order of measurements by counterbalancing) in order to reduce secondary and error variance.

Descriptive analyses (mean and standard deviation for continuous variables; n and percentage for categorical variables) will be conducted for all the study variables. 

To address the first research question, exercise behaviour and HAPA constructs of the motivational and volitional phases (primary outcomes) will be compared across different phases (baseline, post-intervention and follow-ups) (H1) and between the different study groups (intervention participants, sedentary controls, active controls) (H2 and H3). Within-group and between-group analyses will be conducted using parametric (e.g., 1-way or 2-way ANOVAs, Student *t* test) or non-parametric tests (e.g., Friedman’s *χ*^*2*^ test and Wilcoxon’s *Z* pair comparisons for related samples, Kruskal–Wallis’ *H* test and Mann–Whitney’s *U* pair comparisons for independent samples). The choice of test will be determined by the compliance with parametric assumptions of normality and homoscedasticity. A calculation of the effect size will be also conducted (e.g., Cohen’s d). Covariates (e.g., age, menopausal symptom burden) will be introduced (e.g., ANCOVAs).

The second research question (H4 and H5) will be addressed using analyses of bivariate associations (e.g., Pearson *r*, Spearman *rho*), multiple linear regression analyses and multilevel clustering analyses to explore the correlates and predictors of outcome variables as well as intraindividual profiles or patterns of associations. Path analyses and analyses of indirect relationships between variables (e.g., mediation, moderation) will allow for a deep understanding of the complex associations between the study variables. Structural equation modelling will allow for testing the fit of data to the model based on HAPA predictions. Covariates will be introduced to control for their confounding effects (e.g., perceived health status). 

In addition, psychometric properties of the measures will be analysed (e.g., Cronbach’s *alpha*, intercorrelations).

A significance level of *p* < 0.05 will be established.

## Discussion

A recent study has concluded that Spain, which is currently the fourth longest-lived country in the world with a life expectancy of 83 years, will be ranked the highest in longevity in 2040, with a predicted increase of approximately 3 to 5 years of life expectancy, depending on living conditions and evolution of health indicators [1]. The study also highlights that the main causes of morbidity and premature death in the coming decades will be linked to non-communicable diseases, particularly those related to cardiovascular and metabolic disorders, overweight and obesity. Moreover, it is emphasised that lifestyle habits and inequalities related to health and disease processes will play a crucial role in shaping global health trends. In the most pessimistic scenarios predicted by the study, life expectancy could potentially decrease by almost half across all countries in the next generation. The findings of this study could potentially guide researchers, professionals and policymakers in making efforts aimed at improving health systems and policies at local, national and global levels. Ultimately, these efforts should be aimed at health promotion, reducing the burden and costs associated with disease at individual and societal levels, as well as promoting a global healthy life trajectory.

Women constitute a population that demand attention from health care systems and policies [107–109]. In particular, women going through the menopause and beyond — which is a natural and universal process associated with ageing — experience a myriad of biological, psychological and social changes in their life trajectories. This subpopulation of women is showing an increasing interest in their own health and well-being [109]. While for many women, the perimenopause and postmenopause are not necessarily associated with complaints and disease burden, there are others for whom menopausal symptoms are so profoundly disturbing that their quality of life is severely reduced [110], particularly when they have additional risk factors such as obesity or cardiometabolic risk [111]. In addition, they may be concerned by long-term health risks [112, 113].

The impact of menopause-related changes on women's quality of life is strongly linked to personal and sociocultural characteristics, which significantly determine how each woman experiences the perimenopausal transition and the postmenopausal stage. A growing body of evidence shows the influence of various psychosocial factors on women's health during and after menopause, including their perceptions, beliefs, attitudes and lifestyle habits [114, 115]. These factors can in turn influence women’s engagement in health promotion and disease management programmes, the development of support networks, the seeking of professional help and the choice and adherence to therapies such as hormone therapy and other interventions, including lifestyle changes [27]. Therefore, psychosocial factors are important precursors of good health among women in midlife. For this reason, it has been suggested that this life stage may have a positive — rather than negative — impact on women [116]. While the link between psychosocial factors and women’s personal experiences of menopausal symptoms and health-related decision-making has been extensively studied, it remains unclear as to how these factors interact with biomedical variables for influencing the choice of intervention strategies that women use to manage both menopause symptoms and their effects in the short and long term.

All the above demonstrates the importance of implementing exercise programmes for postmenopausal women. It is also crucial to identify and manage the psychosocial and biomedical factors that determine the initiation and adherence to an active lifestyle, beyond the benefits for a woman’s health, well-being and quality of life. Particularly with respect to the determinants of exercise behaviour in middle-aged women, there is limited literature that considers the experiences, needs, resources and opportunities of women in the postmenopausal period. The latter is especially important if one considers the low adherence to the recommended guidelines [10] and the dropout rates (i.e., from partial or temporary abandonment to total and permanent disengagement) among those who have participated in an exercise programme, so that up to half of these women fail to adopt the new habit, particularly as they age or face health conditions [117]. Moreover, gender inequalities should guide practical and policy-oriented efforts to eradicate the differences among women and men in terms of health and lifestyle [107].

The findings of the AHAWOMEN study will offer valuable insights into issues of great significance concerning the health and quality of life of postmenopausal women, specifically in relation to the factors involved in the adoption of healthy behaviours, including exercise. This knowledge will enable a deeper understanding of how to design effective interventions tailored to the needs, opportunities, resources, experiences and circumstances of women in middle age. These interventions should incorporate a range of action strategies, with a particular emphasis on behavioural strategies designed to promote positive health behaviours. Thus, the findings of this research can provide the foundations for developing theory-grounded and evidence-based interventions aimed at promoting an active lifestyle among postmenopausal women. These initiatives have the potential to significantly improve the health and well-being of this population, while preventing disease, premature mortality and unhealthy ageing.

This paper presents the original study protocol. Any significant changes to the protocol will be duly reported to the Institutional Ethics Board, included in the ISRCTN Register and described with transparency in subsequent publications.

### Strengths and limitations

The study has several strengths. First, it is based on a strong conceptual framework, using the HAPA model to examine the social cognitive factors and processes involved in health behaviour change. Moreover, the study considers the realities and experiences of the study population to contextualise exercise behaviour, which enhances its ecological validity. Another strength of the study is its multidisciplinary and synergistic nature, involving experts from various fields including Health Psychologists, Clinical Psychologists, Exercise & Sport Psychologists, Gynaecologists and Sport & Exercise Physicians, which broadens the scope of the procedures and results. In addition, the focus on middle-aged women’s health and well-being contributes to both existing knowledge and its social applicability.

An important limitation of the study concerns the sample size. However, we believe that the chosen sample is justified for two reasons. First, it is necessary to include participants with three different levels of physical behaviour (non-active, initiators and regularly active). Second, the study involves an exhaustive exploration of the variables of interest in each evaluation phase, which will not be feasible with a larger sample as this would hinder the optimal monitoring of participants. In addition, as mentioned, the expected sample size per study subgroup exceeds the calculated sample size.

Further, it is worth noting that our study primarily relies on self-reported data. However, previous research has demonstrated the reliability, validity and accuracy of self-reports for gynaecological data [118], psychosocial data [119], behavioural data [120] and physical activity data [121, 122], while both objective and subjective methods have been shown to offer convergent yet complementary information. Given that single item measures generally perform more poorly than multi-item questionnaires [123], we prioritised the use of multi-item questionnaires in our assessment protocol.

In addition, there is a lack of previous research on the fit of the HAPA model for predicting active behaviour in early postmenopausal women [64, 65, 68]. In order to more thoroughly explore the influences of HAPA constructs on exercise behaviour adoption, longer interventions (i.e., exercise programme, HAPA-based intervention) and follow-ups would be necessary. Nevertheless, 3 to 4 months is considered to be sufficient to progress through the intention to action phases, while an additional 12-month period allows for the transition from initiation to the initial sustainability of habits, taking into account the natural fluctuations in behaviour [50, 124–128]. Evidence suggests that the main motivational and self-regulatory changes that improve adherence occur in the first 1–2 months of practice [129] and the first 6 to 12 months of adherence [130], respectively.

Finally, the type of methodology used in the design and data analysis (i.e., methodological and statistical control) will make it possible to overcome the limitations of the study design and its potential weaknesses. Nonetheless, further research will be needed to replicate our findings.

## Conclusions

In conclusion, the findings of the AHAWOMEN project will provide valuable insights for designing and implementing effective theory-grounded and evidence-based interventions. The midlife stage appears to be a crucial period for interventions aimed at promoting exercise and an active lifestyle, since the ageing population becomes less active — particularly women —, the ageing processes become more evident and health threats and chronic diseases begin to appear. Midlife is thus viewed by many as an opportune time to initiate a change in lifestyle. Embracing an active lifestyle after the menopause can have long-lasting benefits, helping women to remain active for years to come while managing the above-mentioned phenomena. All this stresses the importance of promoting exercise programmes specifically tailored to middle-aged women. Since both the initiation and maintenance of an active lifestyle pose significant challenges, it is essential to manage the psychosocial and biomedical factors that determine short- and long-term adherence. To optimise the benefits of exercise for women’s health, well-being and quality of life, it is crucial to consider the needs, opportunities, resources, experiences and circumstances of women in midlife. The AHAWOMEN project, through multidisciplinary alliances and conceptual and empirical contributions transferable to healthcare and social justice, will provide solutions to the main current societal and public health challenges.

The authors plan to disseminate the findings of this study through publications in peer-reviewed journals and by presenting the work at scientific and professional conferences.

## Data Availability

The datasets used and/or analysed during the current study are available from the corresponding author on reasonable request. There will be no personal identification of participants in the data set.
